# Sociocultural factors associated with detection of autism among culturally and linguistically diverse communities in Australia

**DOI:** 10.1186/s12887-023-04236-2

**Published:** 2023-08-23

**Authors:** Aniqa Hussain, James Rufus John, Cheryl Dissanayake, Grace Frost, Sonya Girdler, Lisa Karlov, Anne Masi, Tasha Alach, Valsamma Eapen

**Affiliations:** 1 Discipline of Psychiatry and Mental Health, School of Clinical Medicine, University of New South Wales, Sydney, NSW Australia; 2 Ingham Institute for Applied Medical Research, Liverpool, NSW Australia; 3 School of Psychology and Public Health, Olga Tennison Autism Research Centre, La Trobe University, Melbourne, VIC Australia; 4 Autism Specific Early Learning and Care Centre, Prospect, South Australia Australia; 5 School of Allied Health, Faculty of Health Sciences, Curtin University, Perth, Australia; 6 Cooperative Research Centre for Living with Autism, Brisbane, Australia; 7 Director of Therapy and Clinical Services, Autism Association of Western Australia Inc, Subiaco, WA Australia; 8 Academic Unit of Child Psychiatry, South Western Sydney Local Health District, Liverpool Hospital, Sydney, NSW Australia

**Keywords:** Autism spectrum disorder, CALD communities, Age of first concern, Sociocultural risk factors

## Abstract

**Background:**

The age at which parents or caregivers first develop concerns about their child’s development has significant implications on formal diagnosis and intervention. This study aims to determine the sociocultural factors that are associated with the age and type of first concern reported by parents of autistic children among culturally and linguistically diverse (CALD) communities in Australia. We also assessed whether sociocultural factors predict autism traits measured in terms of social affect (SA), restricted and repetitive behaviours (RRB), and calibrated severity scores (CSS).

**Methods:**

This study is a secondary data analysis of the data collected from six Autism Specific Early Learning and Care Centres (ASELCCs) as part of the Autism Co-operative Research Centre (CRC) program between 2015 and 2019. Data analysed in this study included a family history questionnaire with sociodemographic and sociocultural information, parent-reported age and type of first concern, and clinician/researcher administered Autism Diagnostic Observation Schedule - Second Edition (ADOS-2) which includes standardised domain-wise scores of social affect (SA) and restricted and repetitive behaviours (RRB) as well as calibrated severity scores (CSS), a measure of severity of autism. Primary analysis included multivariable linear regression models to examine the predictive influence of sociodemographic and sociocultural factors on the dependant variables of age of concern (AOC) and the autism traits (SA, RRB, and CSS).

**Results:**

The mean AOC in the sample was 18.18 months and the most common concerns were speech/language delay, limited social interaction, and hyperactivity/behavioural changes. The multivariable linear regression models showed factors such as increase in age of child, those from a CALD background, annual family income, sibling’s autism diagnosis, and developmental concerns to be significantly associated with parental AOC. Additionally, we also found that increase in child’s age and CALD status to be significant predictors of autism trait (RRB) and severity measured in terms of the CSS score. Further, females (compared to males) were associated with higher difficulties with social communication and interaction skills.

**Conclusion:**

Understanding key factors that contribute to early identification of autism can help tailor awareness programs for parents and caregivers, whilst also informing the development of services focused on serving all CALD communities.

## Background

Autism Spectrum Disorder (ASD; hereafter ‘autism’) is a life-long neurodevelopmental condition characterised by social, communication, and behavioural changes ranging in degree and presentation [1]. The global prevalence rate of autism is rising with the current estimate at 1 in 44 children [2]. There is substantial evidence suggesting that early detection and support for autistic children are associated with improved outcomes, with the American Academy of Paediatrics recommending commencing interventions by two years of age [3–8]. For example, a recent systematic and meta-analysis of personalised, non-pharmacological interventions for younger autistic children such as naturalistic developmental behavioural interventions and developmental interventions have shown to be effective forms of early intervention strategies in achieving a range of developmental outcomes [9]. These patient-centred, early assessment and intervention have the potential to leverage the neural plasticity of the developing brain, mitigate regression [10–12], and positively impact developmental outcomes [13, 14].

With the growing interest around early interventions for autism, has come the need to better understand the manifestation of symptoms of autism at a young age. Globally, there are wide disparities in the age of diagnosis of autism. Although some studies have reported that autism can be accurately diagnosed as early as 18 months of age [7, 15], it is not the case in most children, globally. A recent systematic review analysed data from 40 countries and concluded that the average global age of diagnosis of autism was 43.18 months, ranging between 30.90 and 74.70 months [16]. On the other hand, evidence shows that the parental age of first concern (AOC) - the age at which parents or caregivers first develop concerns about their child’s development, has shown to be significantly associated with the age of ASD diagnosis [17, 18]. However, it is important to understand that parental AOC could be influenced by several child-level factors (for example, severity of child’s autism and other co-morbidities) as well as parent-level factors (for example, parental health literacy, socioeconomic status, and cultural influences). Therefore, it is reasonable to consider that there could be several risk factors which might potentially impact the timing and detection of autism. Hence, the identification of key risk factors could provide an opportunity to implement early interventions to enable better outcomes for autistic children and their families – a key objective of this study.

There is a growing body of evidence suggesting that the presentation, interpretation, reporting of autistic traits as well as treatment strategies for autism appear to be susceptible to cultural influences [19, 20]. In simple terms, culture is defined as “a set of behavioural norms, meanings, and values or reference points utilised by members of a particular society to construct their unique view of the world, and ascertain their identity” [21]. Further, the term ‘culturally and linguistically diverse’ is used to describe communities with diverse languages, ethnic backgrounds, nationalities, traditions, societal structures, and religions [22]. What is considered atypical behaviour in Culturally and Linguistically Diverse (CALD) families may differ from families from Western countries, delaying the recognition of autism traits [22–24]. For example, behaviours such as limited eye contact is considered typical behaviour in certain Indigenous Australian and Asian cultures [25, 26] and may therefore go unrecognized as a potential indicator of autism. Whilst initial parental concerns for Hispanic children were more likely to be related to language and speech delay, motor delay and not responding to their name were also more common in Hispanic compared to non-Hispanic children [27, 28]. These cultural differences in presentation can result in delays in autism diagnosis and intervention if early features remain unrecognised.

Differences in autism presentations are only one of the contributing factors to delays in autism diagnosis in CALD populations. However, cultural attitudes towards autistic children may result in delayed intervention, leading to negative health outcomes. An example of this is seen within certain Indigenous communities associate identifying disability with shame, leading to social isolation from family and community. Therefore, the stigma around disability may lead to individuals less likely to seek support and intervention [29]. Subsequently, screening policies and early intervention services are largely not tailored towards the needs of CALD families, with various cultural and linguistic needs, many of whom experience challenges in accessing these services [30, 31]. Therefore, it is critical to understand the interplay of culture in autism since cultural views regarding appropriate behaviours and normal development for a certain culture may impact parent/carer reports, and ultimately influence the timing and nature of autism diagnosis and treatment [19, 32].

Besides cultural differences, the social determinants of negative health outcomes such as health illiteracy, inequitable and inadequate access to healthcare, and socioeconomic status could also influence the timing of autism diagnosis and subsequent access to support [33–35]. A recent Australian systematic review found that these barriers included a lack of accessibility due to cost and availability, acceptability of services due to religious/cultural reasons, and affordability [36]. Further, health literacy is crucial for understanding developmental expectations to assist in early identification of autism. Kelly et al. (2017) found higher rates of autism diagnosis among mothers of children with higher levels of education than those with lower levels of education [33]. In addition to this, Thomas et al. [37] demonstrated that a higher household income is related to higher levels of autism diagnosis. However, there is lack of knowledge on the sociodemographic and sociocultural factors associated with parental age and type of first concern among CALD communities in Australia.

To address this knowledge gap, we aim to explore the relationship between sociodemographic and sociocultural factors, age of first concern (AOC), and core autism symptoms, using a large Australian dataset. It is expected the findings will inform service recommendations for community-wide identification, diagnosis, and interventions for autism with a view of providing early detection and support for multicultural populations.

## Methods

### Aims

The overall aim of this study was to determine the sociocultural factors that are associated with the age and type of first concern reported by parents of autistic children among CALD communities in Australia. Specifically, we sought to identify the average AOC, how types of concerns correlate with autism traits, and the associated sociocultural factors (income, parental employment, CALD background, parental education, family structure, autistic sibling, and country of birth) that predict AOC. We also assessed whether sociocultural factors can predict autism traits measured in terms of social affect (SA), restricted and repetitive behaviours (RRB), and severity of autism based on calibrated severity scores (CSS).

### Study hypotheses

We hypothesise that there is a significant association between sociocultural factors and AOC. Additionally, we hypothesise that key sociocultural factors could also predict autism traits (SA, RRB) and severity (CSS).

### Study Design, study setting, and sample size

This study is a secondary data analysis of the data collected within ‘The Autism Subtyping Project’ which is a multicentre, controlled, pre-post study measuring autism traits, developmental skills, adaptive functioning, and behaviours, both before and after the implementation of an intervention program [38]. This study utilised data collected from six Autism Specific Early Learning and Care Centres (ASELCCs) as part of the Autism Co-operative Research Centre (CRC) program at the time of the child’s entry to the program between 2015 and 2019. These ASELCCs were located in New South Wales, Queensland, South Australia, Tasmania, Victoria, and Western Australia and provided early intensive intervention programs in a long-day care setting and support to the child’s family. Although minor differences existed across all sites in terms of the number of participants which was dependent on the capacity of each ASELCC centre, the core structure, strategies, and processes of selection, recruitment, consenting of participants, and data collection methods were similar across the centres.

The sample size was calculated based on the capacity of the ASELCCs to enrol approximately 20 children on an annual basis in the early intervention program with one centre able to accommodate up to 44 children per year. Over a 6-year period, this yielded a sample size of approximately 750 children which allowed for 90% power, at a two-sided 5% significance level, to detect a 0.25 standardised difference in mean intentions scores between intervention and control group at follow-up whilst allowing for 20% lost to follow-up.

### Participants

The eligibility criteria included families of children enrolled in early intensive intervention programs at one of the six ASELCCs and met criteria for a diagnosis of autism based on the DSM-IV or DSM-5 diagnostic criteria [1] or had features consistent with an autism diagnosis. No other specific inclusion or exclusion criteria and no pre-screening measures were utilised. Participants that met the eligibility criteria were invited to participate in research and gave informed consent if they wished to participate.

### Data collection

Data collection was undertaken by either the manager of each ASELCC or a Research Assistant, who have been trained by experts in the field to become familiar with various sets of data collection forms and assessments. Data collectors also attended formal training to administer the Autism Diagnostic Observation Schedule - Second Edition (ADOS-2) [39]. All outcome measures and questionnaires were either administered or collected at the initial assessment (baseline) and at exit from the centre. Research Electronic Data Capture (REDCap) tools [40, 41] were used for data entry and audit related tasks Further details about data collection of other clinical data in the Autism Subtyping Project is described elsewhere [38].

### Outcome measures

Age of first concern was defined as the age of the child (in months) at which parents or carers first developed concerns regarding their child’s development.

Autism behaviours (traits) were measured via the ADOS-2 [39] a semi-structured, standardised diagnostic observational assessment which was used to confirm diagnosis of ASD. The ADOS-2 was administered by a trained clinician or researcher. The administered module was determined by the child’s age and expressive language ability. Therefore, Module 1 was given to children aged 31 months and older without phrase speech, Module 2 was given to children who were not verbally fluent with phrase speech, and lastly, Module 3 was given to children who were fluent in language.

The ADOS-2 assessment consists of two behavioural domains such as restricted and repetitive behaviours (RRB) as well as social affect (SA) which includes items relating to communication and social interaction. The item scores in these domains are converted to algorithm scores of 0 to 2, with higher score indicating more severe deficits. Based on the algorithm scores of both domains of SA and RRB, the standardised calibrated severity scores (CSSs) are calculated using validated algorithms [39, 42, 43]. The CSS provides a more accurate measure of core autism symptom severity that is relatively independent of child age and other characteristics [42]. Whilst the CSS score provide some advantages over other measures of general ASD severity, the nature of the symptoms underlying an individual’s CSS may vary greatly [43]. Thus, to obtain a clearer picture of ASD dimensions, we used the domain scores of SA and RRB as well as ADOS-CSS as separate models in the multivariable linear regression analyses (see data analysis section).

All of the above outcome measures were completed at baseline (i.e., within eight weeks of beginning the early intervention program at the ASELCC).

### Explanatory variables

The data pertaining to the AOC was part of a family history questionnaire (FHQ) that was completed by the primary caregiver at baseline. The sociodemographic variables considered in this study included child-level and parent/carer-level characteristics. The child-level variables were: gender (male/female); country of birth (Australia/other); CALD status (no/yes); whether the child was a firstborn (no/yes); whether the child had a sibling (no/yes); whether the sibling had a diagnosis of autism (no/yes); and type of first concern (speech/language delay, unresponsiveness, motor signs, restrictive and repetitive behaviour, eye contact, sensory changes, limited social interaction, developmental delay, and hyperactivity/behavioural changes – no/yes). Further, the parent/carer-level variables included education level (primary or secondary/ tertiary or postgraduate education) and occupation (professional or paraprofessional/ other) of primary and secondary carers as well as annual family income (less than $40,000/ $40,001-$85,000/ $85,001-$115,000/ more than $115,000).

### Data Analysis

We used descriptive statistics such as mean and standard deviation to report on data measured on a continuous scale whereas frequency counts and percentages were calculated for categorical measures. The data including dependent variable was checked for normality using the Shapiro-Wilk test and quantile-quantile (Q-Q) plots. Differences in the distribution of the mean AOC according to child-level and parent-level characteristics were tested with independent samples t-test and analysis of variance (ANOVA), and adjusted for multiple comparison with the Bonferroni method. Homogeneity of variances was assessed by the Levene’s test for equality of variances. Cohen’s d and partial eta squared with 95% confidence intervals were used to report effect sizes for independent samples t-tests and ANOVA analyses, respectively.

Our primary analysis included four multivariable linear regression models to examine the predictive influence of sociodemographic and sociocultural factors on the dependant variables of AOC (model 1), autism traits in terms of SA (model 2) and RRB scores (model 3), and autism severity measured as CSS score (model 4). We used the backward stepwise elimination method where variables based on their non-significant p-values were manually removed at each step of the model until all the variables in the final model were significant (p-value < 0.05). Variable selection in the stepwise analyses was directed by the BIC criterion, which is a penalized likelihood model selection criterion used to compare different models. We used the BIC criterion with the stepwise approach because it favours a model that includes the fewest variables and to reduce the risk of false positives inherent to stepwise analysis [44]. For example, for AOC as outcome variable, three models were assessed (baseline model – CALD; baseline model + sociodemographic factors; baseline model + sociodemographic factors + types of concerns). The assumptions for multivariable linear regression analyses were tested using scatterplots and Q-Q plots to confirm the linearity of associations, normal distribution of the residuals, and homoscedasticity. Further, variance inflation factors (VIF) were used to identify multicollinearity and Durbin–Watson tests were calculated to determine autocorrelation. All statistical analyses within this report were conducted using RStudio (version 2022.07.0 “Spotted Wakerobin”) and SPSS Statistics v.27 (SPSS for MacOS, SPSS Inc., Chicago, IL, USA).

## Results

### Descriptive findings

The descriptive characteristics of this study sample can be found in Table 1. About 94% of the respondents who completed the FHQ were mothers who also identified themselves as the primary carer. The mean (SD) age of children at the time of enrolment was 3.43 (0.86) years (i.e., 41.23 (10.36) months). Of the 759 participants in the dataset with recorded gender, 607 (80%) were male and 152 (20%) were female. A majority (84%) of the participants were born in Australia. However, 290 (38%) participants reported as being from a CALD background. The most common type of first concern was speech/language delay (58.2%), followed by limited social interaction (27.8%), and hyperactivity/ behavioural changes (25.3%).

**Table 1:** Child-level and parent-level characteristics of the sample by mean age of first concern

Variables	N (%)	Mean AOC (SD)	Effect size (95% CI)	Adjusted p-value*
**Child-level characteristics**				
Gender			0.24 (0.05, 0.43)	0.24
*Males*	607 (79.9)	18.56 (8.1)		
*Females*	152 (20.0)	16.6 (7.4)		
*Missing*	1 (0.1)			
Country of birth			-0.49 (-0.78, -0.21)	**0.02**
*Australia*	634 (83.4)	17.89 (7.9)		
*Other*	53 (7.0)	21.78 (7.5)		
*Missing*	73 (9.6)			
CALD status			-0.36 (-0.52, -0.21)	**0.02**
*No*	393 (51.7)	16.95 (8.3)		
*Yes*	290 (38.2)	19.78 (7.2)		
*Missing*	77 (10.1)			
Do you have siblings?			0.15 (-0.06, 0.35)	1.00
*No*	121 (15.9)	19.15 (7.5)		
*Yes*	436 (57.4)	18.02 (7.8)		
*Missing*	203 (26.7)			
Sibling with ASD			0.31 (0.12, 0.49)	**0.02**
*No*	315 (41.4)	18.77 (8.1)		
*Yes*	181 (23.8)	16.35 (7.5)		
*Missing*	264 (34.8)			
Firstborn child			-0.18 (-0.33, -0.03)	0.44
*No*	360 (47.4)	17.54 (7.6)		
*Yes*	348 (45.8)	18.93 (8.3)		
*Missing*	52 (6.8)			
Type of concern				
*Speech/Language delay*	442 (58.2)	19.55 (7.4)	-0.47 (-0.63, -0.32)	**0.02**
*Unresponsiveness*	159 (20.9)	17.28 (6.9)	0.15 (-0.03, 0.32)	1.00
*Motor signs*	101 (13.3)	16.71 (6.5)	0.22 (0.01, 0.43)	0.92
*Restrictive and Repetitive Behaviour*	178 (23.4)	17.43 (8.1)	0.13 (-0.04, 0.30)	1.00
*Eye contact*	191 (25.1)	18.09 (7.1)	0.12 (-0.15, 0.18)	1.00
*Sensory changes*	79 (10.4)	16.64 (7.8)	0.22 (-0.02, 0.45)	1.00
*Limited social interaction*	211 (27.8)	19.00 (8.0)	-0.15 (-0.31, 0.01)	1.00
*Hyperactivity/behavioural changes*	192 (25.3)	18.43 (8.1)	-0.04 (-0.21, 0.12)	1.00
*Developmental delay*	114 (15.0)	14.66 (7.9)	0.53 (0.33, 0.74)	**0.02**
**Parent/Carer-level characteristics**				
Education level of Primary carer			-0.07 (-0.24, 0.10)	1.00
*Primary/secondary education*	198 (26.1)	17.75 (8.7)		
*Tertiary/postgraduate education*	483 (63.6)	18.32 (7.7)		
*Missing*	79 (10.3)			
Education level of Secondary carer			-0.06 (-0.23, 0.11)	1.00
*Primary/secondary education*	200 (26.3)	17.72 (8.6)		
*Tertiary/postgraduate education*	397 (52.2)	18.18 (7.5)		
*Missing*	163 (21.5)			
Occupation of Primary carer			0.15 (-0.02, 0.32)	1.00
*Professional/paraprofessional*	188 (24.7)	18.99 (7.8)		
*Other*	471 (62.0)	17.78 (8.1)		
*Missing*	101 (13.3)			
Occupation of Secondary carer			0.08 (-0.09, 0.24)	1.00
*Professional/paraprofessional*	290 (38.2)	18.31 (7.6)		
*Other*	299 (39.3)	17.71 (8.1)		
*Missing*	171 (22.5)			
Annual Family Income			0.05 (0.02, 0.09)	**0.02**
*Less than $40,000*	147 (19.3)	17.74 (8.0)		
*$40,001-$85,000*	162 (21.3)	20.83 (9.0)		
*$85,001-$115,000*	99 (13.0)	16.78 (6.8)		
*More than $115,000*	119 (15.7)	16.56 (6.9)		
*Missing*	233 (30.7)			

*Adjusted p-value obtained using Bonferroni’s correction for multiple comparison

**Fig. 1:**
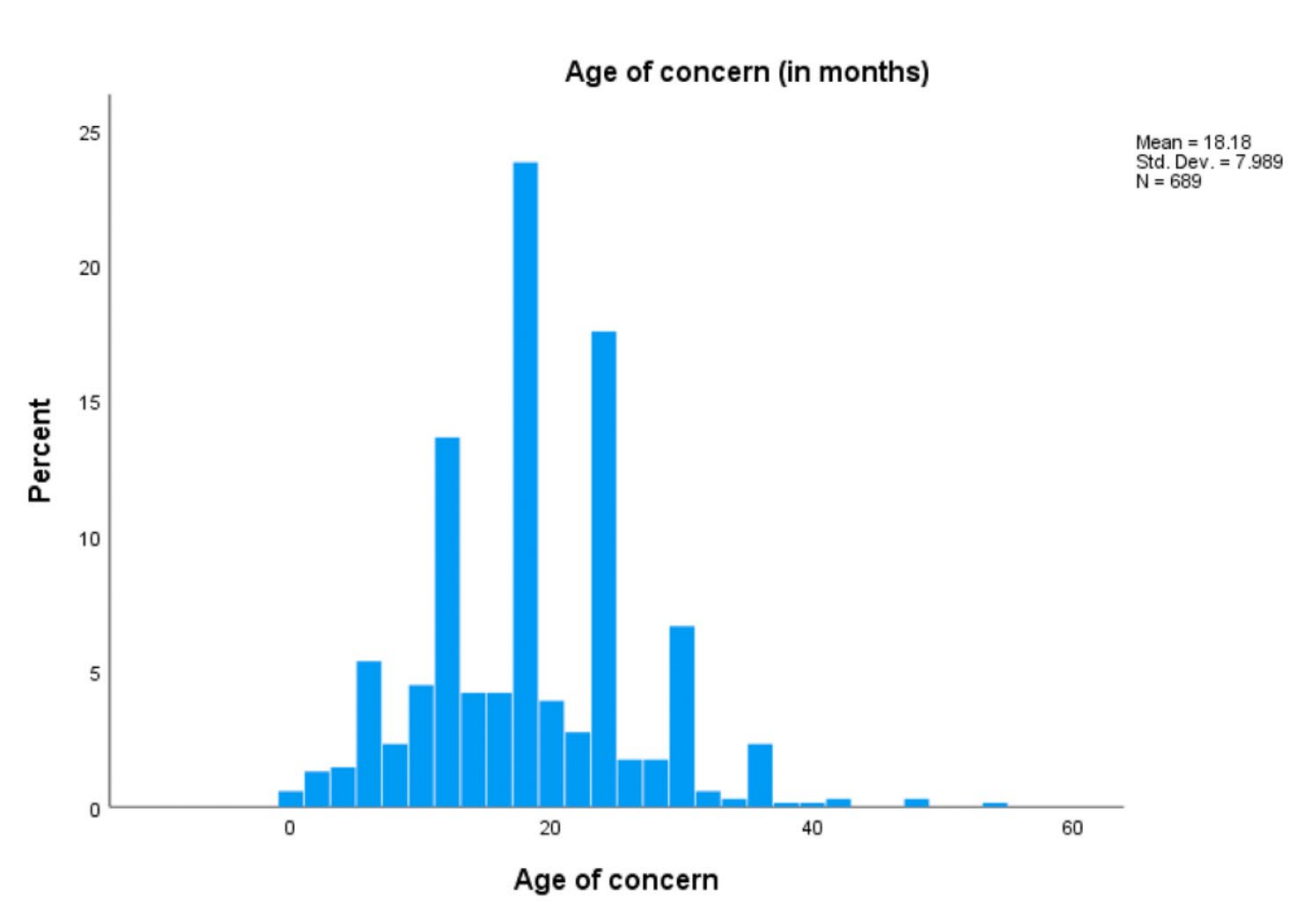
Histogram of age of first concern

The average AOC and ADOS-2 domain scores of the sample are presented in Table 2. It was found that the average AOC was 18.18 months (see Fig. 1). Given the data was collected from a sample of children attending specialised autism early intervention centres, higher scores reflecting higher severity of autism is observed.

**Table 2:** Average age of concern and scores of ADOS-2 domains

Clinical measures	Mean (SD)
Age of concern (months)	18.18 (7.99)
ADOS-2 Social Affect score	14.36 (3.59)
ADOS-2 Restrictive and Repetitive Behaviour score	4.68 (1.97)
ADOS-2 Calibrated Severity Scores	7.16 (1.67)

Findings of the independent t-tests and one-way ANOVA are shown in Table 1. Children born outside of Australia reported delayed AOC of mean (SD) 21.78 (7.46) months compared to mean (SD) 17.89 (7.95) months in those born in Australia, t(664) = -3.37, p = 0.02. Similarly, those from a CALD background were found to have a delayed AOC of mean (SD) 19.78 (7.23) months, compared to those who did not identify as CALD mean (SD) 16.95 (8.27) months, t(660) = -4.60, p = 0.02. Having a sibling with an autism diagnosis resulted in an earlier AOC of mean (SD) 16.35 (7.54) months compared to mean (SD) of 18.77 (8.13) months in those without an autistic sibling, t(483) = 3.24, p = 0.02. The types of concerns most strongly related to AOC were speech/language delay (t(479) = -5.81, p = 0.02) and developmental delay (t(686) = 5.15, p = 0.02) with medium effect size (Cohen’s d ~ 0.50). Lastly, the one-way ANOVA showed statistically significant differences between different levels of annual family income (F(3,510) = 8.78, p = 0.02). Interestingly, a Tukey post hoc test revealed that the mean AOC was statistically significantly lower among families earning highest (more than $115,000) (p = 0.05) and lowest (less than $40,000) (p = 0.05) compared to low to middle income-earning families ($40,001 - $85,000).

### Findings of the multivariable analysis

Four multivariable linear regression models to determine the predictive influence of sociocultural factors associated with AOC, SA, RRB, and CSS (Table 3). Models with the higher adjusted R^2^ and lower BIC values were chosen.

**Table 3:** Multivariable linear regression analyses with AOC, SA, RRB, and CSS as dependent variables

Predictors	Outcome variables			
	Age of concern (AOC) B (95%CI) [p-value] (Model 1)	Social affect (SA) B (95%CI) [p-value] (Model 2)	Restrictive and Repetitive Behaviour (RRB) B (95%CI) [p-value] (Model 3)	Calibrated Severity Scores (CSS) B (95%CI) [p-value] (Model 4)
Intercept	10.45	14.12	6.35	7.42
Age (years)	1.02 (0.54, 1.50) [< 0.001]	NS	-0.45 (-0.64, -0.27) [< 0.001]	-0.24 (-0.40, -0.08) [0.05]
Gender - Females	NS	1.29 (0.43, 2.16) [0.004]	NS	NS
CALD background - Yes	2.15 (0.56, 3.75) [0.008]	NS	0.60 (0.02, 1.17) [0.011]	0.55 (0.40, 1.07) [0.008]
Annual income	-0.94 (-1.63, 0.25) [0.008]	NS	NS	NS
Sibling’s ASD diagnosis – Yes	-1.66 (-3.25, -0.07) [0.041]	NS	NS	NS
Speech/Language delay – Yes	3.21 (1.57, 4.85) [< 0.001]	NS	NS	NS
Developmental delay - Yes	-3.32 (-5.50, -1.13) [0.003]	NS	NS	NS

Primary Outcome (AOC – Model 1) – Cohen’s f^2^ = 0.41; adjusted R^2^ = 0.29; F = 11.62 on 8 and 352 DF; overall p-value < 0.001; BIC of final model = 2509.65 (compared to baseline model = 4623.34 and baseline model + sociodemographic variables = 2528.18)

B – unstandardized b coefficient (slope)

NS – not significant in the final model

In terms of AOC as an outcome variable (primary outcome), an increase in age was significantly associated with a 1.02-month delay in AOC (β = 1.02; 95% CI 0.54, 1.50 at p < 0.001). Compared to children from a non-CALD background, children from a CALD background had a 2.2-month delay in AOC (β = 2.15; 95% CI 0.56, 3.75 at p = 0.008). Having a sibling with an autism diagnosis was significantly associated with an earlier AOC of 1.66 months (β = -1.66; 95% CI -3.25, -0.07 at p = 0.041). Similarly, it was also found that affluent households had a significantly earlier AOC of 0.94 months (β = -0.94; 95% CI -1.63, -0.25 at p = 0.008). For types of concern, whilst presence of general developmental delay was significant associated with earlier AOC by 3.3 months (β = -3.32; 95% CI -5.50, -1.13 at p = 0.003), speech/language delay was associated with a 3.21-month delay (β = 3.32; 95% CI 1.57, 4.85 at p < 0.001) in AOC.

With regards to autism traits and severity, the multivariable linear regression found that female children (compared to males) were significantly associated with increase scores for the SA domain of ADOS-2 (β = 1.29; 95% CI 0.43, 2.16 at p < 0.001). However, with regards to RRB, increase in age was associated with a lower RRB score by 0.45 (β = -0.45; 95% CI -0.64, -0.27 at p < 0.001) whereas those from a CALD background were significantly associated with higher RRB scores by 0.60 (β = 0.60; 95% CI 0.02, 1.17 at p < 0.001) compared to those who did not identify as CALD. This trend was consistent with dependent variables as CSS, measuring the severity of autism (Table 3).

## Discussion

To our knowledge, this is a comprehensive study with a large sample of Australian preschool children receiving intensive early intervention for autism, assessed using standardised measures at entry to the program and post-intervention. We found that the average AOC within our cohort was 18.18 months whereas the average age of diagnosis was 28 months, a delay of 10 months. Although it is expected that there is a 5-to-6-months difference between the initial concern and diagnosis, studies have shown that this delay is on average at least 1.5 years after initial parental concern [45–47]. Therefore, it is thought that several factors may have contributed to the timing of parental AOC and subsequently, delay in autism diagnosis. In particular, we found factors such as increase in age of child, those from a CALD background, annual family income, sibling’s autism diagnosis, and developmental concerns to be significantly associated with parental AOC. Additionally, we also found that increase in child’s age and CALD status to be significant predictors of autism trait (RRB) and severity measured in terms of the CSS score and females to have higher difficulties with social communication and interaction skills as measured by ADOS-SA domain.

In this study, we found significant association between CALD status and delayed age of concern as well as higher severity of autism traits (RRB domain and CSS scores). The main findings are in keeping with our previous research and other Australian study that found evidence for an ‘inverse care law’ in that, children from more disadvantaged backgrounds, including those from CALD background, who are at highest developmental risk, are least likely to access prevention and health promotion programs such as developmental surveillance, and they are late in seeking help for developmental issues [48, 49]. As a result, there is a delay in initiation of intervention due to late diagnosis and thereby missed opportunities to maximise the plasticity of the developing brain [50]. In line with this theory, the delayed AOC and the delay in help seeking behaviour may also have contributed to those with higher severity seeking intervention, in general. This is also reflected in the higher ADOS-2 scores, and specifically higher scores in the RRB domain and the overall severity of autism as measured by the CSS score.

This study also found that a positive diagnosis of autism among siblings can influence parents’ sensitivity to departures from typical development and lead to an earlier age of concern. This is consistent with the findings from previous literature [51, 52] where parents of an older autistic child have increased awareness of early signs and are more likely to scrutinize the younger child’s development. Whilst gender was not a significant predictor of AOC, we found that females (compared to males) were associated with higher scores in the social affect domain. Although in contrary with previous literature which suggest that females have a later age of diagnosis, this seem to suggest that parents of those with more severe clinical features, particularly girls with prominent impairment in social communication and interaction skills may have been the ones who sought intervention from the ASLECCs.

With regards to the initial type of concern, speech/language delay, although reported to be the most common concern (58%), it was found to be associated with delayed AOC by 3.21 months. There is substantial evidence that speech/language delay is often the most commonly reported concerns for early identification of autism [18, 51, 53]. However, the conflicting evidence from this study could be due to the reason that, since speech and language delays are not specific to autism, it could be more easily underestimated by parents with respect to more evident motor impairments [17]. Another plausible reason for the delayed timing of concern related to speech/language delay could be due to cultural beliefs of parents where speech delays are often normalised or overlooked [54, 55].

This study also found that children who initially presented with general developmental delay had an AOC more than three months earlier than average. This finding is consistent with previous evidence [56, 57] and could be due to better parental awareness about general development and related delays and better understanding of expected developmental milestones [58]. Further, general physical symptoms such as a motor skill deficit or feeding difficulty may also be more recognisable by parents as opposed to delays in receptive or expressive communication [17]. Thus, it is essential that screening tools that are sensitive to all aspects of development along with culturally sensitive awareness programs implemented within the health care setting to increase early identification of autism.

High income was also found to be associated with earlier AOC and autism diagnosis within this study. Both high and low prevalence rates of autism diagnosis have been identified based on socioeconomic group status [59–61]. However, the higher documented rates of autism amongst families from higher socioeconomic background may be due to delayed identification or lack of identification of autism in lower socioeconomic communities [59, 61, 62]. Guthrie et al. found autism prevalence rates to be 2–3 times higher in non-Caucasian children and lower-income households compared to Caucasian and higher-income families [60]. However, it has been reported that non-Caucasian children, from lower-income households, who received public insurance and were exposed to a language other than English were less likely to be screened for autism [63]. Therefore, the rates of autism amongst disadvantaged groups have been greatly underrepresented due to the lack of screening. The International Classification of Functioning, Disability and Health indicate that environmental factors contributing to health equity not only includes the attitudes and relationships of those immediately involved in the care of an individual, but also the services, systems, and government policy contributing towards their health [64]. For this reason, it is essential that government funded services increase the available screening and therapeutic opportunities for autism with a focus on minority populations, in order to reduce the current inequity of services available within Australia and expand culturally sensitive programs.

This study has several strengths and limitations. The validity of relationships depicted within this study was strengthened by the size of the ASELCC dataset of 760 children across Australia and this sample size was a notable strength of this study. However, there may have been sampling bias given that the data was collected from children attending specialised autism early intervention centres. It is possible that the children attending the ASELCCs by and large had higher severity of autism. Hence, children continuing to go undiagnosed during the preschool years, or children who were not accessing this specific type of intervention centre were not represented. The generalisability of the finding, then, may be limited. Another limitation of this study was the subjective nature of broadly categorising the initial parental concerns into types of concerns which could have been influenced by observer bias. Further, while this paper primarily focussed on the sociocultural factors, it is possible that other factors including developmental/cognitive level and co-morbidities may have had an impact on the age and type of first concern. Thus, further studies must be conducted to address these limitations for increased clinical significance.

## Conclusion

Identifying early features of autism is critical for providing early intervention as this offers better outcomes. Understanding factors that contribute to early identification of autism can help tailor awareness programs for parents and caregivers, whilst also informing the development of services focused on serving all ethnically diverse communities. The finding that first born children, children born overseas, and children from a CALD background had delayed AOC is indicative of the need for targeted community programs for priority and minority populations about the early signs of autism. These results have international significance in that identifying and addressing contextual and sociocultural factors that influence age and types of first concerns can help tailor community awareness campaigns and population health programs to facilitate early detection and address barriers to early intervention for autism.

## Data Availability

The datasets generated and/or analysed during the current study are not publicly available due to its sensitive nature. However, aggregate data may be available from the corresponding author on reasonable request.

## Abbreviations

ADOS: Autism Diagnostic Observation Schedule
ADHD: attention deficit/ hyperactivity disorder
ANOVA: Analysis of variance
AOC: age of first concern/ age of concern
ASD: autism spectrum disorder
ASELCC: Autism Specific Early Learning and Care Centres
CALD: culturally and linguistically diverse
CSS: calibrated severity scores
DSM: Diagnostic and Statistical Manual of Mental Disorders
REDCap: Research Electronic Data Capture
RRB: restricted and repetitive behaviour
SA: social affect
SPSS: Statistical Package for the Social Sciences
